# Leukemia Inhibitory Factor Receptor Is Involved in Apoptosis in Rat Astrocytes Exposed to Oxygen-Glucose Deprivation

**DOI:** 10.1155/2019/1613820

**Published:** 2019-02-27

**Authors:** Liang Huo, Yuying Fan, Hua Wang

**Affiliations:** Department of Pediatrics, Shengjing Hospital of China Medical University, Shenyang, China

## Abstract

Leukemia inhibitory factor (LIF) and leukemia inhibitory factor receptor (Lifr) protect CNS cells, specifically neurons and myelin-sheath oligodendrocytes, in conditions of oxygen-glucose deprivation (OGD). In the case of astrocyte apoptosis resulting from reperfusion injury following hypoxia, the function of the Lifr remains to be fully elucidated. This study established models of* in vivo *ischemia/reperfusion (I/R) using an* in vitro *model of OGD to investigate the direct impact of silencing the Lifr on astrocyte apoptosis. Astrocytes harvested from newborn Wistar rats were exposed to OGD. Cell viability and apoptosis levels were determined by the MTT (3-(4,5-dimethylthiazol-2-yl)-2,5-diphenyltetrazolium bromide) assay and annexin V/propidium iodide (PI) staining assays, respectively. Apoptosis was further investigated by the TdT-mediated dUTP nick-end labelling (TUNEL) assay. A standard western blotting protocol was applied to determine levels of the protein markers Bcl2, Bax, p-Akt/Akt, p-Stat3/Stat3, and p-Erk/Erk. The cell viability assay (MTT) showed that astrocyte viability decreased in response to OGD. Furthermore, blocking RNA to silence the Lifr further reduces astrocyte viability and increases levels of apoptosis as detected by annexin V/PI double staining. Likewise, western blotting after Lifr silencing demonstrated increased levels of the apoptosis-related proteins Bax and p-Erk/Erk and correspondingly lower levels of Bcl2, p-Akt/Akt, and p-Stat/Stat3. The data gathered in these analyses indicate that the Lifr plays a pivotal role in the astrocyte apoptosis induced by hypoxic/low-glucose environments. Further investigation of the relationship between apoptosis and the Lifr may provide a potential therapeutic target for the treatment of neurological injuries.

## 1. Introduction

Hypoxic-ischemic encephalopathy (HIE) is known to be a major cause of child mortality and disability, but the processes that lead to neuronal apoptosis in HIE are as yet undefined. Previous research has focused on blood flow and vasculature and the effects on neurons [1, 2]; however, interest has grown in the pathophysiology of astrocytes and their role in HIE-related conditions [3]. Astrocytes are the glial cell type present in the greatest numbers within the brain; they repair and maintain brain tissues and provide trophic, structural, and metabolic support to neurons and facilitate formation of neuronal synapses [4–6]. Given the pivotal role of astrocytes in CNS metabolism and glutamate balance, their death or disruption results in damage to the CNS and neuronal cell death [7, 8]. It is recognised that astrocytes are involved in many events resulting from cerebral ischemia and hypoxia-ischemia (HI) [9, 10]. Specifically, early astrocyte death due to hypoxia and ischemia leads to an interruption in key mechanisms, generating greater neuronal apoptosis, which causes larger lesions and disrupts synaptogenesis [11, 12]. In light of their critical role, it is clear that astrocytes offer a robust therapeutic target to minimise damage resulting from cerebral HI.

Leukemia inhibitory factor (LIF) belongs to an interleukin 6 (IL-6) class and is recognised as a neuroprotector with anti-inflammatory properties [13, 14]. Furthermore, acting on macrophages and T-helper cells, LIF induces an anti-inflammatory phenotype [15]. Production of LIF is stimulated by the proinflammatory cytokines IL-6 and tumor necrosis factor-*α*. The beneficial functions of LIF are demonstrable both histologically and functionally, depending on the maturity and the cell type upon which it acts. Activation of the LIF receptor (Lifr), a 190-kD type 1 cytokine receptor located in the nuclei of neuronal cells until injury occurs, initiates upon binding of LIF [16–18]. Protein-receptor binding forms a high-affinity complex that starts the LIF signal through several cellular pathways, including Janus kinase- (JAK-) signal transducer and activator of transcription (STAT), mitogen-activated protein kinase (MAPK), and extracellular signal-regulated kinase (ERK), preserving stroke-damaged brain cells [19]. Additionally, LIF/Lifr binding drives phenotypic alterations in T cells and macrophages to switch to an anti-inflammatory response from the immediate inflammatory response to brain injury, which leads to neurodegeneration [20, 21]. The signal-transducing role of the Lifr is widely recognised, but its molecular and cellular functions remain to be elucidated.

This research aimed to define the role of Lifr in OGD and the mechanism by which it affects hypoxic-ischemic astrocytes. To accomplish this goal, the impact of RNA disruption of Lifr on apoptosis levels in primary rat astrocyte cultures was assessed. To gain deeper insight into the function of the Lifr in astrocytes deprived of oxygen and glucose, expression levels of a range of apoptosis-related proteins were determined. The proteins assessed included Lifr, B-cell lymphoma 2 (Bcl2), Bax, p-Akt/Akt, p-Stat3/Stat3, and p-Erk/Erk.

## 2. Materials and Methods

### 2.1. Primary Culture of Astrocytes

The Local Animal Ethics Committee of China Medical University approved the protocols and procedures for care and use of animals in this research. Primary astrocyte cultures were derived from newborn Wistar rats. Briefly, the cerebral cortices were harvested following hypothermic anaesthetisation and decapitation. After excision of the meninges, the cortical tissue was cut into small pieces and added to culture medium. The tissue suspension was mixed by vortexing and then filtred through nylon mesh filtres, firstly of pore size 80 *μ*m and then 10 *μ*m. The resulting filtrate was further diluted in Dulbecco's minimal essential medium (DMEM) with 7.5 mM glucose and 20% horse serum. The cultures were placed in a humidified incubator at 37°C / 5% CO_2_. Cultures were maintained by exchanging medium with 10% serum on day 3 and thereafter every 3-4 days. Rotary shaking of the culture flasks at 260 rpm facilitated the removal of other cell types. Primary astrocytes were passaged a maximum of three times in culture and used in assays when 80 – 90% confluent. The astrocyte cultures showed a near-uniform immunoreactivity (greater than 95%) to glial fibrillary acidic protein.

### 2.2. Hypoxia and Glucose Deprivation Experiments

Briefly, following a wash with glucose-free culture medium, primary astrocyte cultures were supplied with fresh glucose-free medium in the absence of serum. For the hypoxic conditions, the cells were placed in a humidified Tri-gas incubator (Thermo Scientific, Waltham, MA) for 24 hours, with conditions of 94% N_2_/5% CO_2_/1% O_2_. Following the 24-hour incubation, the cells were returned to standard incubator conditions (see Section 2.1) for an additional 24 hours. Control cells, used in all assays, were maintained in normal incubator conditions and fed with glucose-containing DMEM.

### 2.3. RNA Interference

Transient transfection of small interfering RNAs (SiRNAs; Jima Medicine, Shanghai, China) was conducted using lipofectamine reagent (Invitrogen, Grand Island, NY) on 70% confluent primary astrocytes in six-well plates in accordance with the manufacturer's instructions. The sequences of the SiRNAs used to interrupt expression of Lifr were as follows: sense 5′ GGUGAUCACGAAGUAACAATT-3′ and antisense 5′ UUGUUACUUCGUGAUCACCTT-3′. Western blotting was used to demonstrate effective downregulation of Lifr expression.

### 2.4. Cell Viability and Apoptosis Assays

MTT assays were conducted using a kit purchased from Sigma (USA) to determine cell viability. To determine levels of apoptosis, cells were double-stained with annexin V/PI in accordance with manufacturer's protocols, and cells stained positively were enumerated by flow cytometry (FACSCalibur™, Becton Dickinson, San Jose, CA). Data were analysed by CellQuest™ software (BD Biosciences). To summarise, the apoptosis protocol was conducted on astrocytes harvested in ice-cold PBS and pelleted by centrifugation. Thereafter, cells were rinsed in staining buffer and exposed to 100 *μ*l of buffer containing 5 *μ*l of annexin V-FITC and 5 *μ*g/ml of PI, for 30 minutes at 4°C in darkness. Following incubation, the cells were washed and resuspended in 250 *μ*l of staining buffer prior to flow cytometry analysis using both forward- and side-scatter light. CellQuest software was used to assess the output from 10,000 events. Apoptotic cells are those that exhibit positive annexin V-FITC staining in combination with negative PI staining.

### 2.5. TdT-Mediated dUTP Nick-End Labelling (TUNEL) Assay

The cells were stained with the* In Situ* Cell Death Detection Kit (Cat. No. 11684817910, Roche), in accordance with the manufacturer's recommended protocol. Cell nuclei were stained with 4′,6-diamidino-2-phenylindole (DAPI, Roche), and the cells were visualised by fluorescent microscopy (Olympus iX70). Levels of apoptosis were calculated as a percentage of positive cells per 1000 DAPI-stained nuclei.

### 2.6. Western Blotting

Astrocyte cultures were prepared for western blotting as described by others [22], with 30 *μ*g of protein (unless otherwise noted) being separated by 10% SDS-PAGE. The gel-separated proteins were then electro-blotted to transfer to a polyvinylidene difluoride membrane. The transferred proteins were probed with the following primary antibodies: Anti-LIFR antibody (ab202847, Abcam, 1:1000), Beta Actin Mouse Monoclonal antibody (66009-1-ig, Proteintech, 1:5000), Phospho-AKT (Ser473) (D9E)XP Rabbit mAb (Cell Signaling Technology, 1:2000), AKT (pan) (C67E7) Rabbit mAb (Cell Signaling Technology, 1:1000), Phospho-Stat3 (Tyr705) (D3A7) XP Rabbit mAb (Cell Signaling Technology, 1:2000), Stat3 (D3Z2G) Rabbit mAb (Cell Signaling Technology, 1:1000), Anti-Bcl2 Rabbit mAb (ab196495, Abcam, 1:1000), Anti-Bax Rabbit mAb (ab32509, Abcam, 1:2000), Phospho-p44/42 MAPK (Erk1/2) (Thr202/Tyr204) (D13.14.4E) XP® Rabbit mAb (Cell Signaling Technology, 1:2000), and p44/42 MAPK (Erk1/2) (137F5) Rabbit mAb (Cell Signaling Technology, 1:1000). The membrane was washed and then incubated with the secondary antibodies HRP-conjugated Affinipure Goat Anti-Rabbit IgG(H+L) (SA00001-2, Proteintech, 1:2000) and HRP-conjugated Affinipure Mouse Anti-Rabbit IgG(H+L) (SA00001-1, Proteintech, 1:2000). After another wash, the detection substrate Immobilon™ Western Chemiluminescent HRP Substrate (Millipore) was applied.

### 2.7. Statistical Analysis

Statistical analysis (*t*-test) was conducted using GraphPad Prism V.7.0, from mean data (± SEM) derived from three independent tests. Statistical significance was determined as* P *< 0.05.

## 3. Results

**Figure 1:**
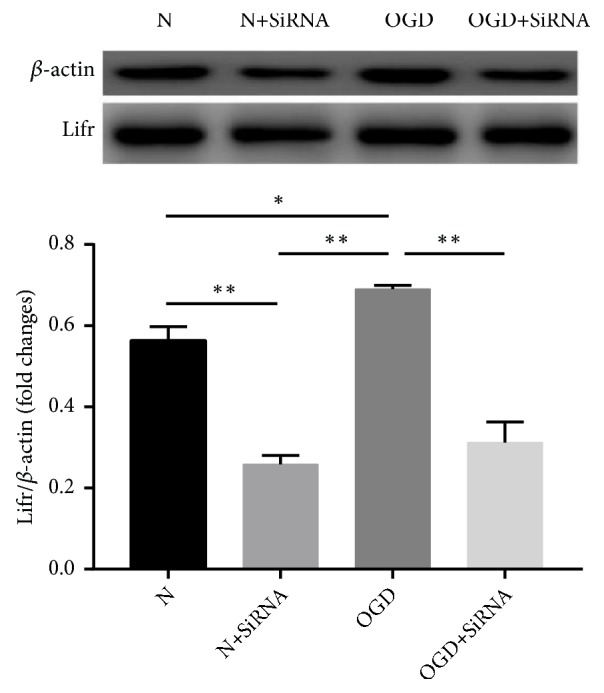
LIFR levels in response to SiRNA treatment in comparison with untreated control and OGD-treated cells. Error bars represent mean ± SEM (*∗P* < 0.05; *∗∗ P* < 0.01).

The efficacy of the Lifr knockdown was measured by western blotting of the LIFR expressed protein. The levels of LIFR were significantly lower (*P* < 0.05) in both the ORNA-silenced groups (N+SiRNA) and oxygen-glucose deprivation (OGD) plus SiRNA (OGD+SiRNA) groups than in either control (normal control, N) or ODG treatment group (Figure 1). Furthermore, the levels of LIFR protein in the OGD treatment group were significantly higher than in the normal control (N) group (*P* < 0.05) (Figure 1).

**Figure 2:**
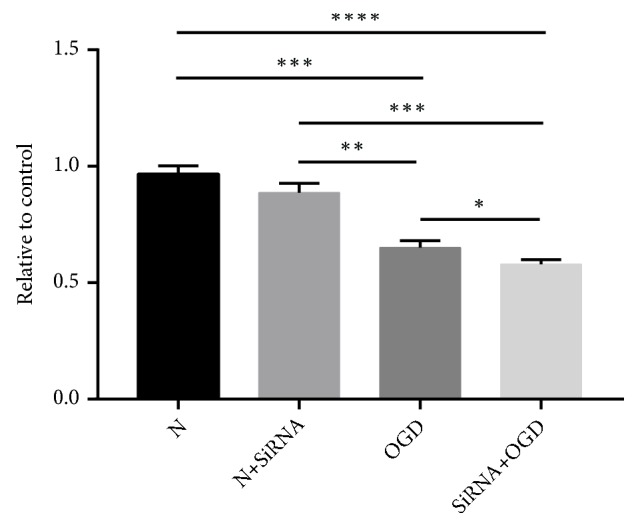
Comparison of cell viability in normal, normal-silenced, OGD-treated, and OGD-treated and silenced astrocytes as determined by MTT assay. Error bars represent mean ± SEM (*∗P* < 0.05; *∗∗ P* < 0.01; *∗∗∗ P* < 0.001).

Astrocyte cell viability was determined by the MTT assay (Figure 2). OGD-treated cells had significantly lower (*P* < 0.01) levels of cell viability, showing a 23% reduction in comparison with the N controls. Silencing of the Lifr in otherwise normal cells led to a slight reduction in cell viability. However, silencing of the Lifr in OGD-treated cells revealed a significant (*P* < 0.05) decrease in viability compared with OGD-treated cells. These data indicate that under OGD conditions, silencing of the Lifr significantly decreased cell viability and stimulated cell damage.

**Figure 3:**
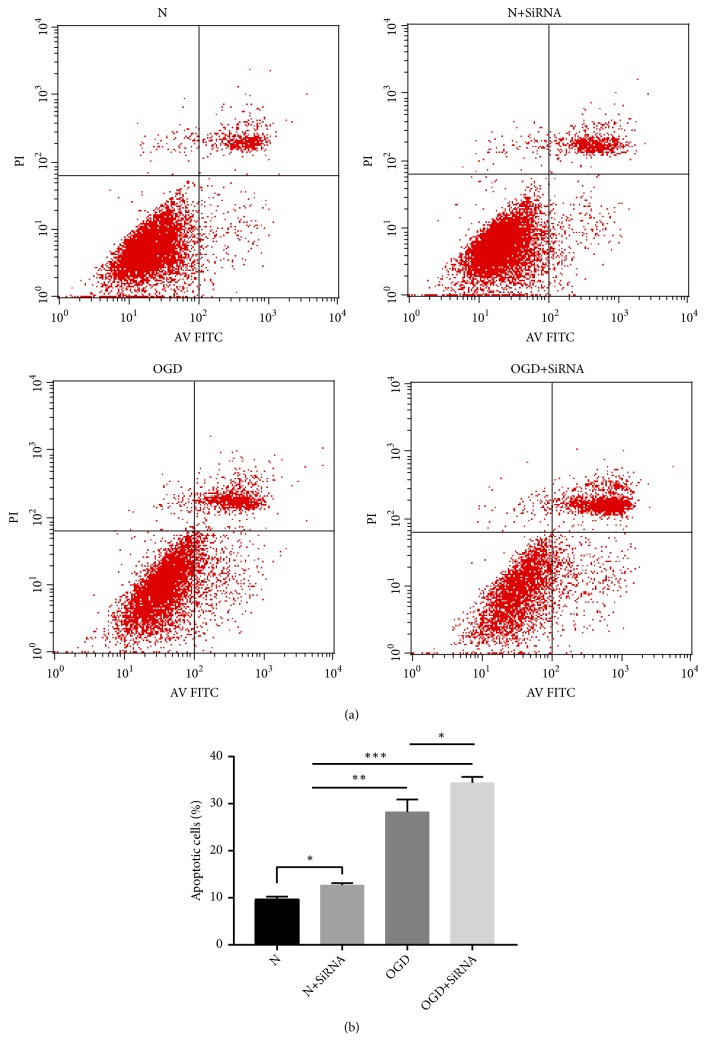
Comparison of apoptosis levels in normal, normal-silenced, OGD-treated, and OGD-treated and silenced astrocytes using annexin V/PI double staining (*∗ P* < 0.05; *∗∗ P* < 0.01; *∗∗∗ P* < 0.001).

Next, we determined the effect of Lifr silencing on apoptosis levels of OGD-treated astrocytes, using the annexin V/PI double-staining method (Figure 3). The results show a significant increase (*P* < 0.01) in apoptosis levels in both OGD-treated and SiRNA+OGD-treated groups in comparison with N and N+SiRNA, respectively. Furthermore, apoptosis increased significantly (*P* < 0.05) in both the silenced (N+SiRNA) group in comparison with the N group and the OGD+SiRNA group in comparison with the OGD group.

**Figure 4:**
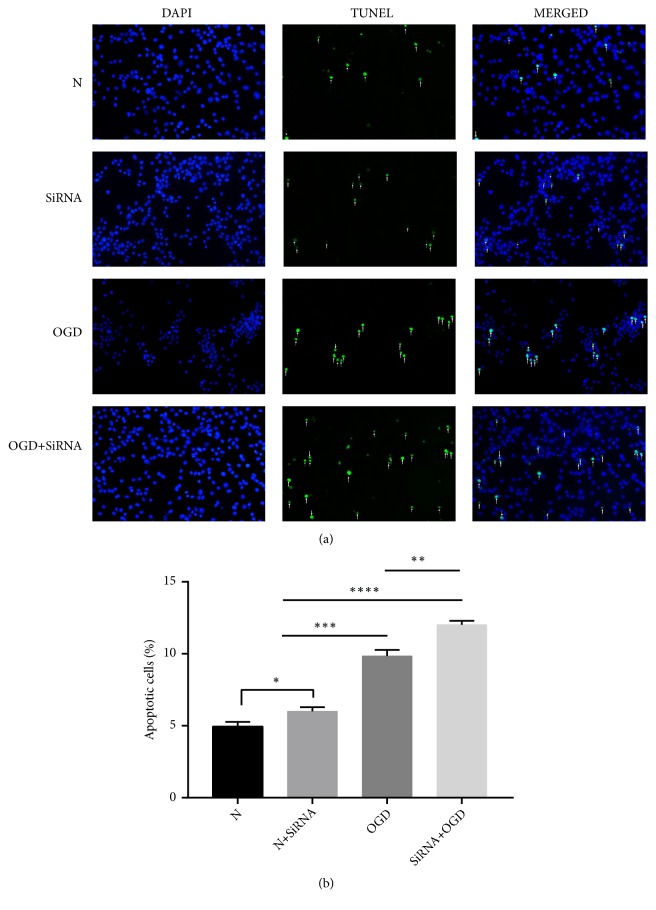
Apoptosis rates in normal, normal-silenced, OGD-treated, and OGD-treated and silenced astrocytes using the TUNEL assay (×200 magnification). Error bars represent mean ± SEM (*∗ P* < 0.05; *∗∗ P* < 0.01; *∗∗∗ P* < 0.001).

Levels of apoptosis were further elucidated by testing each group with the TUNEL assay (Figure 4). N and N+SiRNA astrocytes demonstrated low numbers of apoptotic cells, whereas both OGD and OGD-SiRNA astrocytes exhibited large numbers of apoptotic cells. Silencing of both the N cells (N+SiRNA) and OGD-treated cells (OGD+SiRNA) led to a significant increase in apoptotic cells compared with N and OGD cells, respectively (*P *< 0.05 and* P *< 0.001, respectively). These data suggest that suppression of Lifr further stimulates OGD-induced apoptosis in primary astrocytes.

**Figure 5:**
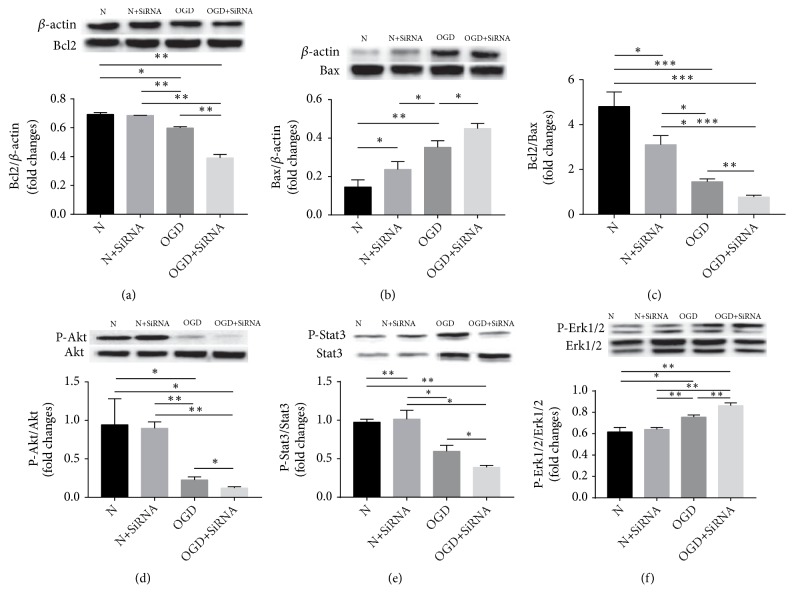
Signaling pathway in OGD-induced apoptosis of astrocytes was detected by western blotting (*∗ P *< 0.05; *∗∗ P *< 0.01).

Levels of proteins in astrocytes that are associated with apoptosis (i.e., B-cell lymphoma 2 (Bcl2), BAX, p-Akt/Akt, p-Stat3/Stat3, and p-Erk/Erk) were assessed by western blotting (Figure 5). OGD treatment led to a significant reduction (*P* < 0.01) in levels of Bcl2 in comparison with the N group, whereas silencing of OGD-treated cells further rescued these levels (*P* < 0.01) in comparison with OGD treatment (*P* < 0.01). Correspondingly, levels of BAX were significantly higher in the OGD group than the N group (*P* < 0.01) and in the OGD-SiRNA group than in the OGD group (*P* < 0.05).

The ratios of the proteins p-Akt/Akt and p-Stat3/Stat3 were significantly lower in the OGD group compared with the N group (*P* < 0.05) and in the OGD-SiRNA group compared with the OGD group (*P* < 0.05). Conversely, ratios of p-Erk/Erk increased significantly in both OGD (*P* < 0.05) and OGD+SiRNA (*P* < 0.01), compared with the N and OGD groups, respectively. In combination, these data indicate a critical role for these pathways in OGD-induced apoptosis, and silencing Lifr may regulate these proteins in a manner that further promotes apoptosis in OGD-treated cells.

## 4. Discussion

The link between brain injury resulting from ischemia and astrocyte damage is widely recognised [23, 24], and it is proposed here that protecting astrocytes in this environment from further apoptotic damage and death may be pivotal in partially protecting brain tissue from ischemic injury and thus improving postischemic functionality. OGD was used* in vitro *to induce apoptosis in astrocytes through two mechanisms: endoplasmic reticulum stress and mitochondrial disruption [25, 26]. Our data show that cell viability, assessed by MMT, is reduced in OGD-treated astrocytes compared with normal (untreated) controls. Furthermore, silencing of the LIF receptor (Lifr) further decreases cell viability and increases apoptosis, as measured using the annexin V/PI method, in comparison with OGD-treated cells. Investigation of apoptosis pathways through western blotting analysis revealed significantly increased levels of BAX and p-ERK/ERK ratios, and a corresponding significant decrease in Bcl2 levels and in p-Akt/Akt and p-Stat3/Stat3 ratios in OGD+SiRNA-treated cells when compared with OGD-treated cells. These data indicate that Lifr control of apoptosis-signaling pathways is important in OGD-stimulated astrocyte apoptosis.

The ratios of Bcl-2 to Bax are intrinsically linked to the balance of apoptosis [27], as Bcl-2 is known as an antiapoptotic protein, the action of which is to prevent cellular apoptosis, whereas Bax is an apoptosis-stimulating protein [27, 28]. A stable ratio of Bcl-2 to Bax has been demonstrated to inhibit H_2_O_2_-induced apoptosis of cultured astrocytes. In apoptotic conditions, the action of Bax after translocation to the mitochondria enables the extracellular release of proapoptotic proteins and operates through permeabilisation of the cell membrane [28].

The protective effects of an increased Bcl-2/Bax ratio, through upregulation of Bcl-2 and corresponding downregulation of Bax, was successfully demonstrated in a coculture of IL-6-producing mesenchymal stem cells and oxygen-glucose deprived astrocytes, wherein the mesenchymal cells successfully inhibited apoptosis [29]. In our study, after OGD treatment, expression of Bcl-2 was decreased in comparison with controls and Bax was correspondingly increased, indicating an increased level of apoptosis. In OGD-treated astrocytes that were also exposed to silencing of Lifr, the ratio of Bcl-2 to Bax further decreased, indicating that, following OGD, suppression of Lifr expression further reduces the Bcl-2/Bax ratio and stimulates apoptosis.

Resulting from cell damage caused by several factors, apoptosis is a widely known cell death mechanism that is initiated by the upregulation of apoptotic protein production [27, 30–32]. Researchers have found that increased levels of Bax expression may lead to apoptosis via activation of procaspase-3 [32, 33], whereas others define the pivotal actions of Bcl-2, p-Akt, and p-ERK1/2 on balancing cell survival and death [34, 35]. Suppression of Akt activity within the CNS is linked to post-HI injury-induced neuronal death, demonstrating the importance of the phosphoinositide 3-kinase (PI3K)/Akt pathway as an antiapoptotic mechanism to protect neurons [36]. The activity of Akt is increased by phosphorylation of serine-473 (Ser473) [37, 38]. We found in this study that p-Akt levels significantly decreased in response to I/R damage, with no effect on levels of unphosphorylated Akt. This response was further enhanced by silencing Lifr, whereby lower levels of p-Akt, corresponding to lower activity of Akt, were found. These data indicate that there is a role for Lifr in apoptosis regulation that may be managed via PI3K/Akt signaling.

Contributing to existing evidence of a neuroprotective role for Lifr, our data elucidate the impact of Lifr silencing on OGD-induced apoptosis. Furthermore, these data enhance our current knowledge of the biological function of Lifr and initiate a pathway for continued research from a clinical perspective to the applicability of Lifr in treating stroke and neurological damage. However, our work investigating the role of Lifr in neurological damage requires further investigation and expansion.

## Data Availability

All the data are available from the correspondence author upon request.
